# mRNA Trafficking in the Nervous System: A Key Mechanism of the Involvement of Activity-Regulated Cytoskeleton-Associated Protein (Arc) in Synaptic Plasticity

**DOI:** 10.1155/2021/3468795

**Published:** 2021-09-23

**Authors:** Michal Fila, Laura Diaz, Joanna Szczepanska, Elzbieta Pawlowska, Janusz Blasiak

**Affiliations:** ^1^Department of Developmental Neurology and Epileptology, Polish Mother's Memorial Hospital Research Institute, 93-338 Lodz, Poland; ^2^Department of Sciences, University of Girona, 17004 Girona, Spain; ^3^Department of Pediatric Dentistry, Medical University of Lodz, 92-216 Lodz, Poland; ^4^Department of Orthodontics, Medical University of Lodz, 92-217 Lodz, Poland; ^5^Department of Molecular Genetics, Faculty of Biology and Environmental Protection, University of Lodz, Pomorska 141/143, 90-236 Lodz, Poland

## Abstract

Synaptic activity mediates information storage and memory consolidation in the brain and requires a fast de novo synthesis of mRNAs in the nucleus and proteins in synapses. Intracellular localization of a protein can be achieved by mRNA trafficking and localized translation. Activity-regulated cytoskeleton-associated protein (Arc) is a master regulator of synaptic plasticity and plays an important role in controlling large signaling networks implicated in learning, memory consolidation, and behavior. Transcription of the *Arc* gene may be induced by a short behavioral event, resulting in synaptic activation. *Arc* mRNA is exported into the cytoplasm and can be trafficked into the dendrite of an activated synapse where it is docked and translated. The structure of Arc is similar to the viral GAG (group-specific antigen) protein, and phylogenic analysis suggests that Arc may originate from the family of Ty3/Gypsy retrotransposons. Therefore, Arc might evolve through “domestication” of retroviruses. Arc can form a capsid-like structure that encapsulates a retrovirus-like sentence in the 3′-UTR (untranslated region) of *Arc* mRNA. Such complex can be loaded into extracellular vesicles and transported to other neurons or muscle cells carrying not only genetic information but also regulatory signals within neuronal networks. Therefore, *Arc* mRNA inter- and intramolecular trafficking is essential for the modulation of synaptic activity required for memory consolidation and cognitive functions. Recent studies with single-molecule imaging in live neurons confirmed and extended the role of *Arc* mRNA trafficking in synaptic plasticity.

## 1. Introduction

Multicellular organisms use several pathways to coordinate cellular processes within specific organs and the whole organism. One of them is cell-to-cell communication over short and long distances. Although the flow of genetic information from DNA to RNA or reversely is restricted to a single cell, both DNA and RNA in their active forms are detected in extracellular fluids, raising the question about their role in cell-to-cell communication and intercellular flow of genetic information [1, 2]. Furthermore, even in a single cell, translation of various mRNAs occurs at specific locations, showing its spatial regulation through mRNA localization [3]. This is especially relevant in highly asymmetric cells such as neurons.

Plasticity in the neuronal network is essential for the ability to learn and keep new information and is underlined by modification of neuronal synapses in response to electrical activity, a process called synaptic plasticity (reviewed in [4]). Long-term memory formation requires gene expression to produce proteins to stabilize fresh changes. The process in which specific synapses are strengthened is termed long-term potentiation (LTP), in contrary to long-term depression (LTD), in which specific synapses are weakened or eliminated. In homeostasis, a high reactivity is required for upcoming activity-dependent synaptic plasticity. Synaptic plasticity may be associated with changes in synapse size, formation of new synapses, or elimination of existing ones (reviewed in [5]).

**Figure 1:**
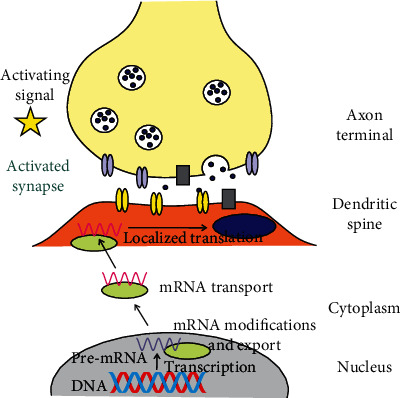
Localized expression of genes is crucial for activity-dependent synaptic modification. Synapse-activating signal (yellow star) may belong to a wide range of stimuli, including behavioral experience. Activated synapse sends a signal to the nucleus to transcribe a gene whose product is needed to modify the synapse. Pre-mRNA is exported to the cytoplasm and can be transported to the site of synaptic modification, where it is translated to produce a protein (dark blue oval) needed for synaptic modification. Alternatively or concomitantly, mRNA may be translated immediately after export to the cytoplasm and the product of translation is transported to the target site (not shown). Light green oval symbolizes all proteins that are needed for pre-mRNA maturation, export, and transport. Neurotransmitters (blue dots) are encapsulated in synaptic vesicles and released into synaptic cleft and are taken up by their receptors (yellow ovals); voltage-gated channels and neurotransmitter reuptake pumps are presented as dark grey box and light blue ovals, respectively.

When activity-induced synaptic modification requires the synthesis of a new protein, it will need a specific mRNA at the site of modification. Therefore, a signal from an activated synapse to the nucleus is needed to induce transcription of the corresponding gene. Then, a localized movement of mRNA or the product of its translation back to the site of modification is required (Figure 1) [6]. Such localized gene expression should occur immediately after activity-dependent synaptic signaling. The expression of the gene encoding the activity-regulated cytoskeleton-associated protein (Arc, Arg3.1, and KIAA0278), an immediate early gene (IGE), represents a mechanism of activity-dependent synaptic modification [6]. Arc is one of the most extensively studied molecules involved in memory consolidation [7]. It is an important effector of the brain-derived neurotrophic factor (BDNF) as well as the glutamatergic, dopaminergic, and serotonin signaling [8]. *Arc* mRNA is transported to the dendrites that are enriched in Arc [6].

Changes in Arc have been associated with several mental disorders, including major depressive disorder, psychosis, and schizophrenia [9–11]. Although a cause-effect relationship has not been established for most of these disorders, Arc is considered a therapeutic target and a prognostic marker in some of them (reviewed in [12]). Arc lowered the synaptic strength in diseases of cognition [13]. Arc expression was impaired in fragile X mental retardation syndrome and metabotropic glutamate receptor-mediated long-term depression (mGluR LTD) [11]. Reduced ubiquitination of Arc may occur in Angelman syndrome [14]. Several reports show an association of Arc with Alzheimer disease (AD) [15–17]. Arc increased the interaction between *γ*-secretase and trafficking endosomes that processed the amyloid precursor protein to A*β* peptide, leading to amyloid deposition [18]. Apart from the nervous system, Arc is also involved in the regulation of the immune response [19]. It is specifically expressed in skin-migratory dendritic cells and regulates their migration and T cell activation [20].

The majority of synapses in the central nervous system are glutamatergic, and they are important sites for environment-dependent plasticity, learning, and memory [21]. Fast excitatory neurotransmission is mediated by N-methyl-D-aspartate receptors (NMDARs) and *α*-amino-3-hydroxy-5-methyl-4-isoxazolepropionic acid receptors (AMPARs) [22]. NMDARs are essential for synaptic development and plasticity [23]. At most excitatory synapses, the expression of NMDARs occurs before AMPARs in early development [24]. Synapses without AMPAR expression are termed “silent” as they are activated only when the cell is depolarized to abolish voltage-dependent block on NMDARs [25]. These synapses are targeted by AMPARs in development, resulting in a decreasing number of silent synapses in the adult brain [26].

In this manuscript, we present the general properties of the Arc protein and *Arc* gene, the mechanisms of *Arc* transcription, and the pioneering works of Steward and his coworkers on the involvement of *Arc* mRNA trafficking in neurons [6, 7, 27–33] as well as the groundbreaking publications of Ashley et al. [34] and Pastuzyn et al. [35] showing intercellular *Arc* mRNA trafficking. The latter is discussed in the context of retrovirus-like sequences in the human *Arc* gene and in the colonization of the human genome by such sequences and their domestication to adjust them to human development.

## 2. mRNA Trafficking in Somatic Cells

Messenger RNA is an intermediate in the expression of protein-coding genes. Transcription of such genes results in the synthesis of pre-mRNA, which is then spliced, edited, end-modified, and transported into the cytoplasm where it undergoes translation to form a polypeptide, which is then proceeded to become a functional or structural protein. However, current studies on the structure of human genes and the human transcriptome have made some modifications to this picture—mRNAs produced in transcription and posttranscriptional modifications are not homogeneously distributed in the cytoplasm, and this fact can be important for cellular functions and energy use.

Subcellular localization of a protein may be reached by a mechanism dependent on the signal peptide (localization sequence) within this protein [36]. The other mechanism is ensured by the selective movement (trafficking), anchoring (docking), and localized translation of mRNA, which is crucial in many processes, including patterning of embryonic axes, asymmetric cell division, cell migration, and synaptic plasticity [35]. mRNA trafficking is especially important in highly asymmetric cells, including neurons. Several mRNAs were shown to specifically localize in axons, dendrites, and cell protrusions [37]. Small fraction of the mRNA pool in mammalian cells may be involved in transfer between cells [38].

**Figure 2:**
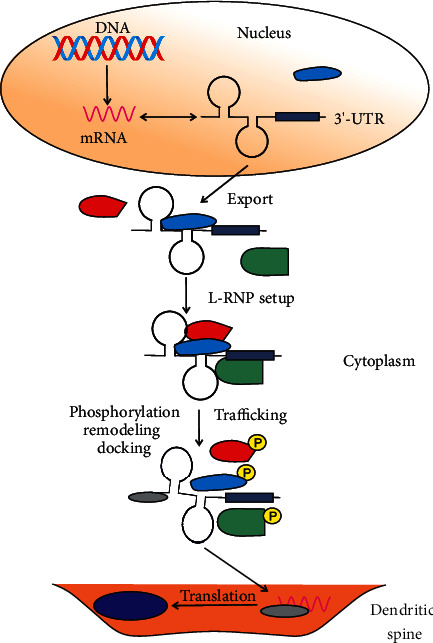
mRNA-protein and protein-protein interactions in localized mRNA expression in neurons. An mRNA resulting from the transcription of a gene in DNA is represented by its 3′-UTR (untranslated region) containing a segment of the specific sequence (filled box) and a higher order structure, represented by two stem loops. These elements handle localized mRNA expression and are recognized and bound by specific proteins (represented by a light-blue object) and exported into the cytoplasm. Then, other RNA-binding proteins, represented by red and green objects, bind to the mRNA 3′-UTR to form a localizing ribonucleoprotein (L-RNP), which is assisted by other proteins (not presented here) and supports the trafficking of mRNA to specific locations in the dendritic spine where it is translated. Before that, mRNA can be remodeled (symbolized by changes in its secondary configuration and protein (grey oval) binding), docked in the dendrite spine, preceded by phosphorylation (the P letter in a yellow circle) and disassociation of the L-RNP proteins, and translated to produce the Arc protein (dark blue oval).

A general pathway of intracellular mRNA trafficking in somatic cells includes complexing of mRNA molecules with proteins in the nucleus, their export to the cytoplasm, and attaching to microtubules or microfilaments [39] (Figure 2). mRNA trafficking involves two distinct stages: mRNA transport and a docking process mediating its selective localization [32]. Many proteins can be involved in mRNA localization within neurons, including Staufen, Bruno, Bicaudal D (BicD), Pumilo (Pum), fragile X mental retardation protein (FMRP), and the Exon Junction Complex (EJC) (reviewed in [40]). Although many genetic aspects of mRNA localization are known, the mechanism behind this effect is not fully known. Its essential step is the recognition of the target transcript depending on short *cis*-elements and localization elements (LEs, zip codes) in the 3′-UTR (untranslated region) of mRNA [41]. Some exceptions include LEs in the 5′-UTR or the coding region of the transcript [42, 43]. Both the sequence and higher-order structure of such elements may be specifically recognized [44] (Figure 2). LEs are targeted by RNA-binding proteins (RBPs) facilitated by a RBP domain recognizing a specific element in mRNA or recruiting auxiliary proteins in complexes called ribonucleoproteins (RNPs or mRNPs). mRNAs are actively transported along the cytoskeleton by specialized motor proteins (dynein or kinesin) or myosins [45]. Proteins that facilitate mRNA trafficking are called mRNA granules. Therefore, localized mRNA translation requires assembling a localizing mRNP (L-RNP) that is sensitive to signals initiating translation in different regions of the cytoplasm [46]. To initiate translation, L-RNP is remodeled by phosphorylation. Some mRNAs do not require transport but only docking in cytoskeleton proteins—actin or intermediate filaments [47]. However, localized mRNA expression may occur even with a homogenous distribution of mRNAs in the cytoplasm by the suppression of translation or degradation of the translation product in nontarget locations. Another possibility is that a protein can be distributed uniformly in the cytoplasm and selectively captured in a specific site.

## 3. Arc: An Immediate Early Gene and a Master Regulator of Synaptic Plasticity

*Arc* is a single-copy gene expressed in the cortical and hippocampal glutamatergic neurons. It is highly conserved in vertebrates and expressed in the nucleus, dendrites, and postsynaptic density [8]. The Arc protein is a master regulator of synaptic plasticity, involved in learning, memory consolidation, and behavior.

### 3.1. Arc: The Gene and the Protein

The *Arc* gene is located at 8q24.3 in minus strand, and its most recent chromosomal allocation is 142,611,049-142,614,479 (GRCh38/hg38) (https://www.genecards.org/cgi-bin/carddisp.pl?gene=ARC&keywords=arc, accessed August 10, 2021) [48].

Arc was discovered in searching for IEGs that responded to neuronal activity independently of protein synthesis. Arc is unique among other IEGs, as it is not a transcription factor and immediately moves through the dendritic arbor of the neuron in which it is activated—it was shown that after a single electroconvulsive shock, Arc was distributed in the molecular layer of the dentate gyrus, containing the dendrites of the dentate granule cells, while mRNAs of other IEGs were in the cell body [33].

Arc is involved in the regulation of endocytosis of AMPARs [49–51] (reviewed in [52]), Notch signaling [53], and spine size and type [54].

### 3.2. Arc Expression

*Arc* transcript appears within 5 minutes of induction, making it a genuine IEG [55]. The transcription of the *Arc* gene is induced by a variety of factors, including seizures in the hippocampus, enhanced neuronal activity in response to learning, LTP, and LTD (reviewed in [56]). In normal conditions, *Arc* is transcribed at low levels, which may be further decreased by AMPARs, which can be considered a stabilizer of *Arc* transcription [57]. The extracellular-signal-regulated kinase (ERK) is a central hub of signaling pathways downstream of several receptors whose activation is essential for a sudden increase in *Arc* transcription. These receptors include group 1 metabotropic glutamate receptors (mGluR1s), BDNF tropomycin-receptor kinase (Trk) B, muscarinic acetylcholine receptors, and NMDARs [32]. ERK phosphorylates coactivators of the serum response factors (SRFs), such as Elk-1, which is a ternary complex factor, binding to serum response element (SRE) genes in their promoters to activate transcription. *Arc* has a major SRE-responsive region in its promoter located about 6.5 kb upstream of its transcription start site (TSS) [58]. The other major SRE is about 1.4 upstream of TSS. However, the involvement of these elements in *Arc* transcription regulation has not been definitely confirmed [59]. A 6.9 SRE was reported to be essential for LTD in cultured Purkinje cells [60].

**Figure 3:**
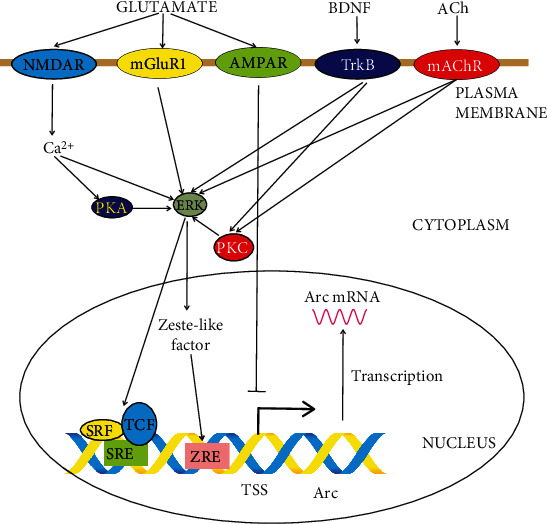
Regulation of the transcription of the activity-regulated cytoskeleton-associated protein (Arc) gene. Some known and putative pathways are presented only. Basic low-level Arc transcription can be further decreased by the activation of *α*-amino-3-hydroxy-5-methyl-4-isoxazolepropionic acid receptor (AMPAR). Arc transcription is promoted by signaling through the NMDAR (N-methyl-D-aspartate receptor), mGluR (group 1 metabotropic glutamate receptor), TrkB (tropomycin-receptor kinase B), and mAChR (muscarinic acetylcholine receptor) signaling pathways through the interaction with several downstream kinases, including ERK (extracellular-signal-regulated kinase), PKA (protein kinase A), and PKC (protein kinase C). ERK increases Arc transcription through its coactivator TCF (ternary complex factor) binding to SRE (serum response element) in the Arc promoter. ERK may also increase Arc transcription through its Zeste-like factor directly interacting with ZRE (Zeste-like response element) in the Arc promoter. The transcription start site (TSS) and direction of transcription are marked by the bold arrow above the Arc gene. Ach: acetylcholine; BDNF: brain-derived neurotrophic factor.

Several other regions in the *Arc* promoter have been identified or suggested to play a role in *Arc* response to neuronal activity, including a region 1.4 kb upstream of TSS containing a Zeste-like response element, a site binding to myocyte-enhancer factor-2 (Figure 3) [56].

Before translation, *Arc* mRNAs are exported into the cytoplasm and then moved in the target sites. The association of *Arc* mRNA with the kinesin motor complex and the maximal speed of its movement (over 60 *μ*m/min) suggest its active transport [27]. The ERK signaling is necessary for LTP-induced translation of *Arc* mRNA. It is initiated through the MAP kinase-interacting kinase (MNK) and phosphorylating eukaryotic initiation factor 4E (eIF4E) [61].

### 3.3. Arc in Synaptic Plasticity and Cognition

Arc may be implicated in learning and memory as they both are underlined by synaptic plasticity (reviewed in [62]). A positive correlation between *Arc* mRNA expression in the rat hippocampus and spatial learning in the Morris water maze was observed [63]. In addition, an increased concentration of *Arc* mRNA was measured in the cytoplasm, but not in the nucleus. Behavioral experience that activates the synapse may belong to a wide range of tasks, including those as diverse as sound exposure and complex reversal learning.

Two phases of *Arc* expression were shown in the CA1 hippocampal neurons during fear memory formation in mice [64]. An initial transient *Arc* expression just after the stimulus was observed in the first phase and a second increase in Arc levels after 12 h, depending on BDNF and essential for memory consolidation. A similar delayed expression of *Arc* was observed during sleep-dependent consolidation of cortical plasticity in the cat visual cortex [65]. Therefore, a transient *Arc* induction may occur in two-phase induction of expression during memory consolidation. In line with this suggestion are results with *Arc*-knockout mice showing that Arc might be needed for long-lasting memory for implicit and explicit learning tasks, despite intact short-term memory [66]. The *Arc*-knockout animals exhibited biphasic changes of hippocampal LTP in the dentate gyrus and the CA1 area with an increased early and absent late phase. Furthermore, LTD was considerably impaired in that experiment. These results confirm an essential role of Arc in the consolidation of enduring synaptic plasticity and memory storage, although the mechanism eliciting such delayed reactivation is still unclear.

A more robust expression of *Arc* in rat cortical regions was observed after recalling a recent (24 h after training) memory relative to remote (1 month after training) memory [67]. An increased *Arc* expression in the striatum of rats that underwent training/pseudotraining on an operant lever-pressing task was observed [68]. Furthermore, a negative correlation was observed between levels of *Arc* mRNA in specific brain regions of newly trained rats and the rate of task acquisition [69]. This association between Arc and behavior that induces learning suggests that Arc expression, and primarily its transcription, is regulated by signals coming from patterns of neuronal activity. Arc also enables consolidation of weak memories and was shown to play a role in behavioral tagging in the hippocampus [70, 71].

A role of Arc in cognitive flexibility was suggested based on results showing a strong positive correlation between *Arc* mRNA levels in the rat hippocampus and behavioral performance during spatial reversal tasks [63].

Arc was reported to act as a postsynaptic mediator of activity-dependent elimination of synapses in the developing cerebellum of rats by mediating the removal of excessive fiber synapses [72]. Arc targets inactive/weak synapses via a high-affinity interaction with calcium/calmodulin-dependent protein kinase II beta (CaMKII*β*), which is not bound to calmodulin [73]. This interaction may prevent undesired activation of weakened synapses in potentiated neurons. Therefore, Arc may be required to eliminate undesired synaptic material.

A relationship was observed between the overall *Arc* mRNA immunofluorescence signal in the dorsomedial stratum and the number of trials in rats, which underwent a reversal training, but not in rats that experienced continued-acquisition training [74]. This study suggests a differential processing of *Arc* mRNA in different populations of striatal efferent neurons.

Altogether, these results suggest a feedback-like relationship—*Arc* expression may be regulated by large networks linked with learning and memory on the one hand, but on the other, *Arc* expression may form new and modify existing networks.

## 4. *Arc* mRNA Trafficking within Neurons

Synapse-specific gene expression, which is essential for activity-dependent synaptic modification in synaptic plasticity, involves localized translation of specific mRNAs at postsynaptic sites on dendrites [75]. Steward et al. were the first to show that *Arc* mRNA was selectively delivered to postsynaptic sites to mediate synapse-specific gene expression ([7] and reviewed in [6]). These authors also showed that the signals for *Arc* mRNA trafficking resided in mRNA and not in proteins. Moreover, a high-frequency activation of synapses of the perforant path induced *Arc* expression and newly synthesized *Arc* mRNA trafficking to the synaptically activated dendritic laminae. Arc synthesis and *Arc* mRNA localization were observed as separated events. Finally, it was shown that the localization of *Arc* mRNA in activated dendritic laminae was linked with a local accumulation of the Arc protein. In their next work, Steward and Worley showed that the activation of the NMDA receptor was needed for *Arc* mRNA trafficking to active synapses [32]. The Arc protein was assembled into the matrix of the synaptic junction complex.

Newly synthesized and matured *Arc* mRNA is exported to the cytoplasm and actively moves to its target sites [68]. *Arc* mRNA is associated with the kinesin motor complex and other proteins that collectively form the *Arc* messenger ribonucleoprotein complex [76]. This context includes fragile X mental retardation protein (FMRP) and Pur-alpha that both prevent the translation of *Arc* mRNA. The coding region of the *Arc* gene contains the A2 mRNP binding site—the 11 nt A2 response element (A2RE), which is also targeted by CArG box binding factor A (CBF-A), controlling *Arc* mRNA localization by a mechanism regulated by NMDARs and AMPARs [77]. It was shown that a reorganization of the actin cytoskeleton was needed for *Arc* mRNA localization as this process marked synapses for *Arc* mRNA to localize [30].

The newly made Arc protein is quickly localized near active synapses but is also distributed throughout dendrites [6]. Activated dendritic laminae, where *Arc* mRNA migrated, contained accumulated Arc proteins [6]. Arc was assembled into the postsynaptic density (psd)/NMDA receptor complex (NRC) [7]. Further migration of newly synthesized *Arc* mRNA was prevented by its capturing by active synapses. In LTP, *Arc* mRNA and Arc protein localized near active synapses but were also found in other dendritic locations [33].

As mentioned, *Arc* mRNA trafficking is supported by the specific elements in the 3′-UTR. Dynes and Steward showed that a fusion construct containing 3′-UTR of *Arc* mRNA bound by proteins of the coat of the MS2 phage, a single-stranded RNA virus, precisely localized in a microdomain at the base of dendritic spines [28]. That localization was independent of translation, suggesting the presence of a microdomain at the dendritic spine base where *Arc* mRNA was docked. The presence of a dedicated microdomain for *Arc* mRNA docking and translation at the entry to the spine confirms the suggestion that the newly synthesized proteins in dendrites may act preferentially or even exclusively at a single, neighboring synapse [78].

Translation of *Arc* mRNA at specific locations in synapse requires the presence of the ribosome and other components of the translation machinery in these sites. It was assumed that the translational apparatus was closely associated with psd as it synthesized the compounds needed for cotranslational assembly of psd/NRC [7]. However, enhanced levels of *Arc* mRNA in dendrites were not associated with increased levels of exon junction complex proteins [79].

When *Arc* transcription is induced by synaptic activation, *Arc* mRNA is transported to the active parts of dendrites where it is docked for translation. Therefore, two synaptic signals, for transcription and docking, may interplay, but the relationship between them is not fully known [6]. It is not clear whether the capturing signal occurring after LTP lasts enough to capture all mRNAs that came to the active part of synapse, or it is so short that some *Arc* mRNAs may escape docking unless activation signal is kept on. The selectivity of *Arc* mRNA may be also supported by degeneration of the transcript in the nonactive sites, a process called activity-dependent *Arc* mRNA degradation. It was shown that Arc degradation occurred throughout dendrites and required the activation of NMDA receptor and active translation [29]. Such activity- and translation-dependent mRNA degradation was likely specific for *Arc* mRNA as another dendritic mRNA was not degraded in response to synaptic activation. It was shown in another study that such degradation might occur by nonsense-mediated mRNA decay (NMD) as *Arc* mRNA had a splice junction in the 3′-UTR, downstream of the stop codon [80]. This splice junction is involved in the loading of eIF4A3 (eukaryotic translation initiation factor 4A3) onto *Arc* mRNA. Knockdown of *eIF4A3* increased synaptic strength and GLUR1 AMPA receptor load at synapses. Moreover, depletion of eIF4A3 increased Arc, so *Arc* mRNA was a natural target for NMD. Steward et al. using transgenic mice confirmed that *Arc* mRNA degradation occurred with features typical for NMD, and prior splicing of the 3′-UTR influenced fast dendritic delivery of newly synthesized *Arc* mRNA [31].

It is important to determine how long Arc transcription lasts after a stimulating signal. The forebrain neurons, including hippocampal pyramidal neurons, rapidly terminate *Arc* transcription after an inducing event, but dentate granule cells show a continuous transcription of the *Arc* gene [81].

Recent technological developments allow studying mRNA localization and its local translation through the simultaneous detection of mRNAs and proteins in single cells through single-molecule imaging in real time (reviewed in [82]). This has opened new perspectives in the research of memory-associated mRNAs in living neurons in response to neuronal activity (reviewed in [83, 84]). An elegant work from the Singer's and Park's laboratories has substantially progressed our knowledge on the role of *Arc* mRNA trafficking in synaptic plasticity [85]. A mouse model with the *Arc* gene tagged in 3′-UTR with stem loops binding to bacteriophage PP7 coat protein (PCP) was used in that work to visualize single mRNAs in real time. That model allowed following actual dynamics of *Arc* mRNA from transcription to degradation. A robust *Arc* transcriptional burst with prolonged ON state occurred after stimulation in cultured hippocampal neurons. Furthermore, transcription cycles continued after removal of initial stimulation. The correlation of stimulation with *Arc* transcription and mRNA transport in single neurons showed that stimulus-induced Ca^2+^ activity was needed but not sufficient for inducing *Arc* transcription and that inhibiting neuronal activity did not change the dendritic transport of newly produced *Arc* mRNAs.

Fujita et al. used reduction-triggered fluorescent probes combined with fluorescence correlation spectroscopy and observed in real time that *Arc* mRNA was produced after synaptic activation [86]. Analysis of observations confirmed that free diffusion is not enough to transport RNAs of sizes close to *Arc* mRNA. Na et al. showed that glutamate-induced *Arc* mRNA translation was associated with a reactivation of stalled polyribosomes [87]. Moreover, the *Arc* open reading frame was necessary and sufficient for glutamate-induced mRNA Arc translation.

In a more general context, Bauer et al. showed that the molecules of Rgs4 (regulator of G protein signaling 4) mRNA patrolled dendrites to transiently interact with multiple synapses, in agreement with the sushi-belt model [88, 89]. Moreover, the authors observed that the majority of RNA-transporting granules showed bidirectional transport in dendrites independently of 3′-UTR, but Rgs4 3′-UTR caused an anterograde transport bias requiring the Staufen 2 protein. Interestingly, Koltun et al. showed that the level of mRNA translation is similar in neuronal somata and processes and was negatively correlated with the developmental age of living neurons. To show that they used transgenic mice with a reporter gene under the control of the Arc promoter [90], analysis of mRNA trafficking with single-molecule methods in living neurons confirmed and extended the role of *Arc* mRNA trafficking in synaptic plasticity and opened new perspectives in neurosciences [91].

## 5. Intercellular mRNA Trafficking and Signaling

Myrum et al. demonstrated that Arc had two domains on either side of the central linker region [92]. They also showed that Arc is a modular, pyramid-like-shaped protein with a defined secondary structure in its monomeric form. However, such a form was capable of reversible self-association, resulting in large, soluble oligomers. The *Arc* gene has a DNA sequence, which is normally found as its RNA counterpart in retroviruses, such as human immunodeficiency virus (HIV), enabling it to form virus-like particles [93]. These two features are the capacity to self-oligomerize and form virus-like particles which underline Arc ability to enclose molecules, such as RNA or/and protein, and transfer across the synapse to accomplish sending genetic information between neurons [34, 35]. Arc was the first protein to confirm earlier considerations on the role of extracellular vesicles (EVs) in the intercellular communication within the nervous system.

The involvement of Arc in the intercellular sending of genetic information is closely associated with its molecular architecture also found in proteins of some retroviruses. Zhang et al. reported that the crystal structure of Arc formed a bilobar architecture and the N-terminal lobe bound to CaMKII and TARP*γ*2 (transmembrane AMPAR regulatory protein gamma-2, CACNG2, and stargazin) [13]. The domain that mediated Arc binding was structurally similar to the capsid domain of HIV. Further studies revealed that the bilobar configuration of Arc was like the HIV GAG (group-specific antigen) protein. Phylogenic analysis suggested that Arc might originate from the family of Ty3/Gypsy retrotransposons. The members of this family are ancient forms of RNA-based elements able to replicate and be present in animals, plants, and fungi that might be ancestral to modern viruses and certain cellular genes (reviewed in [94]). Therefore, Arc might evolve through the “domestication” of Ty3/Gypsy with the acquisition of the N-terminal coil-to-coil and loss of RNA-binding domain and reverse transcriptase [93].

The role of the GAG-like sequences in *Arc* is not completely clear. In retroviruses, the GAG proteins multimerize during replication to form a capsid that binds to viral RNA and undergoes envelopment by membranes before exiting the host cell in an EV [95, 96].

Extracellular vesicles play a critical role in the communication between neurons, astrocytes, and microglia in normal and diseased brains [97]. In a landmark study, Ashley et al. found that the *Drosophila* Arc1 protein formed capsid-like structures that bound to a specific retrovirus-like region in the 3′-UTR of its own transcript [34] That Arc1-*Arc1* mRNA complex was loaded into EVs and transferred from neurons to muscles. It was not clear whether those vesicles contained multiple or single enveloped capsid-like structure(s). That transfer depended on a specific sequence in the 3′-UTR of *Arc1* mRNA containing retrotransposon-like sequences as disruption of that sequence inhibited the transfer. Transfer of Arc1 and/or *Arc1* mRNA was underlined by mechanisms similar to retroviruses and retrotransposons and was necessary for synaptic plasticity.

At the same time, Pastuzyn et al. showed in another groundbreaking paper the involvement of mammalian Arc in interneuron *Arc* mRNA trafficking [35]. They largely confirmed the results obtained by Ashley et al. and showed that Arc self-assembled into virus-like capsids that encapsulated RNA needed for proper capsid formation.

**Figure 4:**
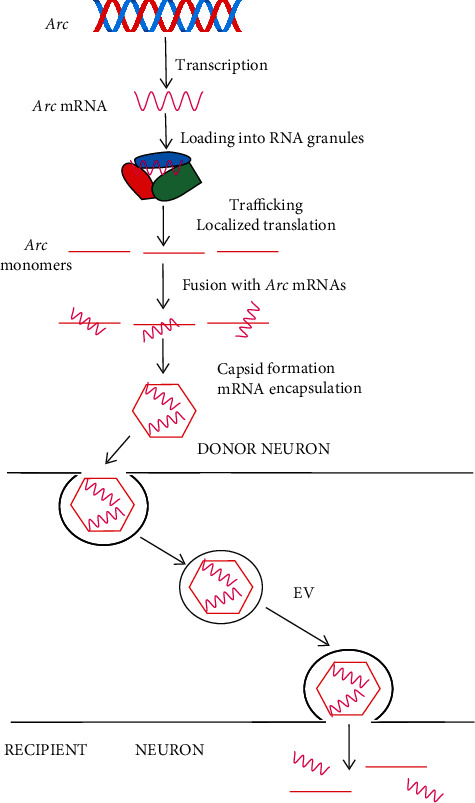
Interneuron transport of activity-regulated cytoskeleton-associated protein (Arc) and its mRNA. Transcription of the Arc gene produces Arc mRNA, which is trafficked to dendrites in RNA granules and translated in response to synaptic activity, resulting in a high local concentration of the Arc protein (red bar), which may oligomerize to form a capsid encapsulating Arc mRNAs. Arc capsid with loaded Arc mRNA departs from the dendrite in extracellular vesicles (EVs) and may be endocytosed by dendrites of different neurons. This may change synaptic activity in response to synaptic activation in another neuron. This figure is limited to Arc mRNA, but RNA granules may contain a selection of different RNAs, and Arc capsids may encapsulate different RNA species and other putative factors. Such transfer was also shown between neurons and muscle cells.

Arc was released from neurons in EVs that mediated the transfer of *Arc* mRNA to target neurons, where it could undergo activity-dependent translation (Figure 4). Arc capsids can be endocytosed, and *Arc* mRNA can be released from EVs into the cytoplasm and trafficked into the target sites in recipient neurons. The interaction between GAG and RNA in viruses is controlled by host proteins, including Staufen that is involved in regulating *Arc* mRNA trafficking in neurons [98]. Therefore, Pastuzyn et al. confirmed that Arc had similar molecular properties as the retroviral GAG proteins. These authors also provide arguments supporting the hypothesis that Arc was evolutionarily derived from a vertebrate lineage of Ty3/gypsy retrotransposons, which are retrovirus precursors. The authors' findings suggest that GAG retroelements have been evolutionarily repurposed to serve in intercellular communication in the nervous system.

## 6. Conclusions and Perspectives

Although *Arc* mRNA was shown to localize to the dendritic spine after high-frequency stimulation of the perforant path synapses over 20 years ago, real-time imaging in awake animals has not been possible [7, 99]. Recent development of single-molecule imaging methods in living neurons opens new perspectives to directly link the role of *Arc* mRNA transport with brain functions and behavior.

One of the most intriguing aspects of the Arc protein is its evolutionary history and a close relation to viruses. It may be speculated that contemporary Arc joins the metazoan genes of viral origin with essential physiological functions. The results obtained by Ashley et al. and Pastuzyn et al. are not limited to *ARC* mRNA [34, 35]. The only requirement seems to be the virus-like sequence within RNA to be trafficked. Therefore, Arc-dependent intercellular RNA transfer may influence many cellular processes, as many various RNAs can be trafficked. The significance of this paradigm is supported by the fact that the human genome contains about 100 genes with homology to GAG and over 150 *GAG* open reading frames encoded as endogenous retroviruses [100]. Therefore, other proteins may form a capsid and traffic their own mRNA or other RNAs. Altogether, the role of intercellular communication underlined by the evolutionary retroviral relics in the human genome in human physiology and disease is likely underestimated, and new studies are needed to shed light on this problem.

Both intra- and intercellular *Arc* mRNA trafficking may play an essential role in synaptic plasticity. There is a natural connection between intra- and intracellular *Arc* mRNA trafficking: intracellular transfer of mRNA leads to its translation in the target sites in dendrites resulting in a high concentration of Arc, which supports capsid assembly in these locations and encapsulation of dendritically localized *Arc* mRNA, which can be then loaded in EVs. Therefore, a potential coordination of intra- and intercellular *Arc* mRNA trafficking requires the precise spatial and temporal expression of *Arc* at the protein level [35]. The question is when such coordination of these two kinds of *Arc* mRNA trafficking is needed, i.e., whether intercellular trafficking is initiated by the *Arc* transcription by a signal coming from a recipient neuron or it is started using Arc and *Arc* mRNA “resources” in the donor neuron. A behavioral experience in a specific location could induce a cascade of intra- and intercellular *Arc* mRNA trafficking in remote cells, resulting in synaptic activity in target location. However, such putative mechanism raises some questions. The experiments of Pastuzyn et al. show that Arc capsids or extracellular vesicles can mediate transfer of *Arc* mRNA between neurons; they do not allow distinguishing Arc, which has been just translated from the Arc protein that was taken up. Secondly, the coordination pathway of such a chain of intra- and intercellular transporting events seems to be very complex. Finally, intercellular transport of *Arc* mRNA encapsulated in *Arc* virus-like capsid is evident in *Drosophila*, but in mammals, the picture is not so clear. Therefore, the question is whether such mRNA transport based on the virus-like structures is evolutionarily appropriate for contemporary mammals. Maybe extensive development of the peripheral nervous system is to compensate for a slow decline in intercellular mRNA transport in mammals. These and many other questions should be addressed in perspective studies on the role and mechanism of *Arc* mRNA trafficking.

As pointed out by Pastuzyn et al., many questions on intercellular *Arc* mRNA trafficking are still open, including except for Arc and *Arc* mRNA “passengers” of EVs docking mechanisms for EVs and spatial/temporal specificity of trafficking in the brain [35]. Both Ashley et al. and Pastuzyn et al. showed that trafficking *Arc* mRNA is available for translational machinery, but the question is whether it could be a substrate for reverse transcriptase by analogy to retroviruses. Therefore, both intra- and intercellular *Arc* mRNA trafficking is essential for the role of Arc in synaptic plasticity and in consequence learning, memory consolidation, and behavior, but further studies are needed to directly show the association between *Arc* mRNA trafficking and behavior or brain function. Finally, the involvement of *Arc* mRNA trafficking in synaptic plasticity is an outstanding example of integrating neuroscience with (retro)virology at the molecular level.
